# Propyl-2-(8-(3,4-Difluorobenzyl)-2′,5′-Dioxo-8-Azaspiro[Bicyclo[3.2.1] Octane-3,4′-Imidazolidine]-1′-yl) Acetate Induces Apoptosis in Human Leukemia Cells through Mitochondrial Pathway following Cell Cycle Arrest

**DOI:** 10.1371/journal.pone.0069103

**Published:** 2013-07-26

**Authors:** Chandagirikoppal V. Kavitha, Mridula Nambiar, Pavan B. Narayanaswamy, Elizabeth Thomas, Ujjwal Rathore, Channapillekoppalu S. Ananda Kumar, Bibha Choudhary, Kanchugarakoppal S. Rangappa, Sathees C. Raghavan

**Affiliations:** 1 Department of Biochemistry, Indian Institute of Science, Bangalore, Karnataka, India; 2 Department of Studies in Chemistry, University of Mysore, Mysore, Karnataka, India; Univ of Bradford, United Kingdom

## Abstract

**Background:**

Due to the functional defects in apoptosis signaling molecules or deficient activation of apoptosis pathways, leukemia has become an aggressive disease with poor prognosis. Although the majority of leukemia patients initially respond to chemotherapy, relapse is still the leading cause of death. Hence targeting apoptosis pathway would be a promising strategy for the improved treatment of leukemia. Hydantoin derivatives possess a wide range of important biological and pharmacological properties including anticancer properties. Here we investigated the antileukemic activity and mechanism of action of one of the potent azaspiro hydantoin derivative, (ASHD).

**Materials and Methods:**

To investigate the antileukemic efficacy of ASHD, we have used MTT assay, cell cycle analysis by FACS, tritiated thymidine incorporation assay, Annexin V staining, JC1 staining and western blot analysis.

**Results:**

Results showed that ASHD was approximately 3-fold more potent than the parent compounds in inducing cytotoxicity. Tritiated thymidine assay in conjunction with cell cycle analysis suggests that ASHD inhibited the growth of leukemic cells. The limited effect of ASHD on cell viability of normal cells indicated that it may be specifically directed to cancer cells. Translocation of phosphatidyl serine, activation of caspase 3, caspase 9, PARP, alteration in the ratio of BCL2/BAD protein expression as well as the loss of mitochondrial membrane potential suggests activation of the intrinsic pathway of apoptosis.

**Conclusion:**

These results could facilitate the future development of novel hydantoin derivatives as chemotherapeutic agents for leukemia.

## Introduction

The growing understanding of the molecular events underlying the etiology of different cancers, as well as the signaling events which are critical for the continued growth and proliferation of cancer cells have enhanced the opportunities to develop novel drugs. Leukemia is one of the major types of cancers which affect a significant segment of the population, especially children [1]. Despite the recent advances and tremendous efforts to improve therapy, the spectrum of available effective drugs is comparably limited and there is a considerable need for the development of new drugs and treatment alternatives. In this regard majority of the research has been focused on developing small molecules that act as anticancer agents, which significantly influence and shape current tumor chemotherapy.

Most cancer therapy drugs induce apoptosis to achieve therapeutic efficacy. The relationship between apoptosis and cancer has been emphasized, with increasing evidence suggesting that the related processes of neoplastic transformation, progression and metastasis involve the alteration of normal apoptotic pathways. In this respect, different apoptotic pathways leading to cytotoxicity have been studied extensively for many compounds [2], [3]. These studies have become a focus of cancer chemotherapy and would shed light on the mechanism of action of candidate drugs. Since, defects in apoptotic pathways such as receptor- and mitochondrial- mediated pathway are the main reasons for the treatment failures in leukemia patients, there is an urgent need to identify the compound which induces apoptosis in leukemia cells.

**Figure 1:**
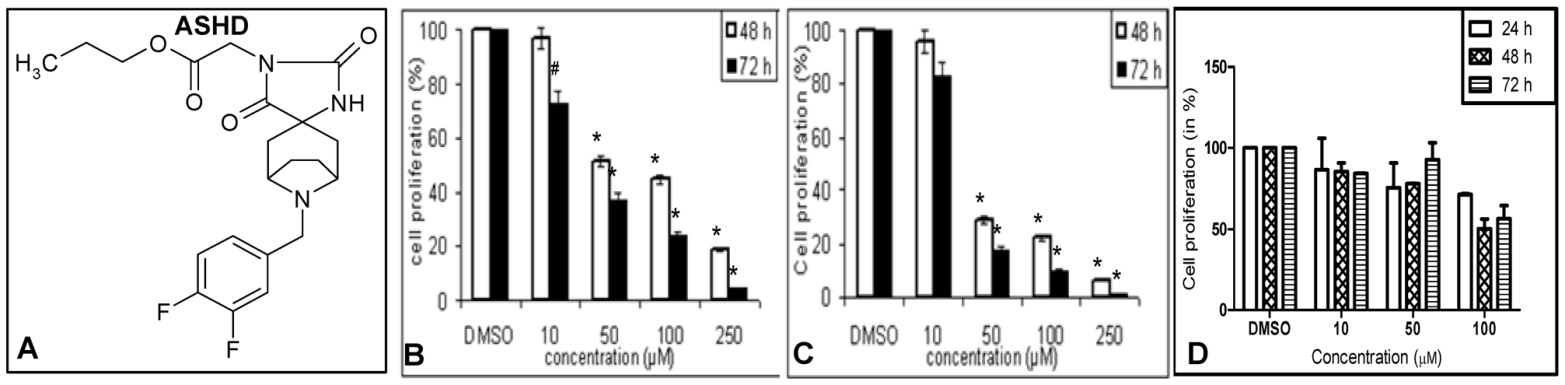
Dose- and time-dependent effect of ASHD on viability of leukemic cells. **A**. Chemical structure of azaspiro hydantoin derivative, ASHD. **B–D**. MTT assay showing effect on cell proliferation following treatment with ASHD. After 48 and 72 h of treatment with ASHD (10, 50, 100 and 250 µM), cells were incubated with MTT to determine cell proliferation. Bar diagram showing percentage of cell proliferation of Reh (**B**), K562 (**C**) and 8E5 (**D**). Error bars represented in each panel is based on three independent batches of experiments.

Hydantoin derivatives possess a wide range of important biological and pharmacological properties [4]–[10]. This pharmacophore is found in a variety of anticonvulsant drugs. In addition, they are being explored as aldose reductase inhibitors, antiarrhythmics, antimicrobials, CGRP receptor antagonists, and anticancer agents [11], [12]. Previously, we have reported synthesis and characterization of a series of hydantoin derivatives [11], [13]. Here, we show that the compound ASHD, an alkyl chain ester group containing hydantoin derivative (Fig. 1), can induce cytotoxicity in leukemia cells with remarkably high efficiency. Treatment with ASHD led to a transient cell cycle arrest at S and G2/M phases, which was confirmed by the observed alteration in CDK2 and cyclin B1 levels. Further, by using various cellular and subcellular assays, we found that ASHD triggers apoptosis through the mitochondrial pathway by altering BCL2/BAD ratio along with the activation of caspases, cleavage of PARP and elevation in the levels of p53.

## Materials and Methods

### Chemicals and reagents

Unless otherwise mentioned, all the chemicals used were from Sigma-Aldrich (USA) or Amresco (USA). Tritiated thymidine ([^3^H] thymidine) was purchased from BRIT (India). Annexin V-FITC and antibodies were purchased from Santa Cruz Biotechnology (USA).

### Synthesis and characterization of spiro hydantoin derivatives

The synthesis of azaspiro bicyclo hydantoin derivatives was described earlier [13]. The formation of the hydantoin ring was confirmed by ^1^H NMR and IR spectra (Fig. 1) [13].

### Cell lines and culture conditions

Human leukemia cell line, K562 and normal human T lymphocyte cell line, 8E5 were obtained from National Centre for Cell Science, Pune (India) and Reh cell line [14] was obtained as a gift from Prof. Michael Lieber, University of Southern California, Los Angeles (USA). All the cell lines were cultured in RPMI1640 (Sera Lab, UK) containing 10% FBS (Gibco BRL, USA), 100 U of Penicillin G/ml and 100 μg of streptomycin/ml (Sigma-Aldrich, USA) at 37°C and 5% CO_2_.

### MTT assay

Cytotoxic effect of ASHD on Reh and K562 cells was assessed using 3-(4, 5-dimethylthiazol-2-yl)-2, 5-diphenyl tetrazolium bromide (MTT) assay. Cells were seeded in duplicates in 96-well plates at 1×10^4^ cells/well. After 24 h, ASHD was added at a concentration of 10, 50, 100 or 250 μM. Following 48 or 72 h of treatment with the compounds, cells were collected and treated with MTT as described earlier [15]. In the case of 8E5, cells were harvested after 24, 48 and 72 h of ASHD treatment (10, 50 and 100 µM). The percentage of proliferating cells was calculated and depicted as a histogram.

### [^3^H] Thymidine incorporation assay

DNA synthesis was monitored by labeling the cells using [^3^H] thymidine. Cells (Reh and K562) were seeded in duplicates in 96 well plates at a volume of 0.125 ml (1×10^5^ cells/ml) followed by the addition of ASHD at concentrations of 10, 50, 100 and 250 μM. After 8 h of treatment, an aqueous solution of [^3^H] thymidine (1 μCi) diluted with RPMI 1640 was added to each well. Following 48 h of incubation, the cells were pelleted, washed and processed as described earlier [16]. Radioactivity of the samples was expressed as disintegrations/min, which was proportional to the amount of [^3^H] thymidine incorporated into the DNA.

### Detection of mitochondrial transmembrane potential (MTP, Δψ_m_)

The changes in the mitochondrial potential were detected by 5,5′,6,6′-tetrachloro-1,1′,3,3′ tetraethylbenzimidazolylcarbocyanine iodide/chloride (JC-1), a cationic dye that exhibits potential-dependent accumulation in mitochondria, indicated by fluorescence emission shift from red (∼590 nm) to green (∼525 nm). MTP assay was carried out as described earlier [17]. In brief, Reh cells (0.75×10^5^ cells/ml) were treated with 10, 50, 100 and 250 μM of ASHD for 24, 48 and 72 h. Cells treated with 4 mM of 2,4-Dinitrophenyl hydrazine (Sigma-Aldrich, USA) for 24 h were used as a positive control. After treatment, cells were stained with JC1 and analyzed by a fluorescence-activated cell sorter (FACscan, Becton Dickinson, USA). The ratio of mean fluorescence intensity (MFI) of red to green fluorescence was calculated for each treatment and plotted.

### Flow cytometric analysis

Distribution of cells (Reh or K562) in different phases of cell cycle was analyzed following treatment with ASHD by flow cytometry as described earlier [17]. Cells were harvested after 72 h of treatment (10, 50, 100 and 250 μM), processed and used for acquiring flow cytometer reading (FACScan, BD Biosciences, USA). A minimum of 10,000 cells were acquired per sample and histograms were analyzed by using WinMDI 2.8 software.

### Annexin-V/PI double-staining assay

In brief, ASHD treated (50, 250 μM, for 72 h) K562 or Reh cells (∼5×10^5^) were harvested, resuspended in binding buffer, stained with Annexin V-FITC and propidium iodide and subjected to flow cytometric analysis as described [15].

### Confocal microscopy

Annexin-V and PI double-stained cells were prepared as described above. Cells were mounted on glass slides using cover slips and viewed under an inverted Zeiss confocal laser scanning microscope (Ziess Meta 510 LSM; Carl Zeiss, Jena, Germany).

### DNA damage analysis by comet assay

Comet assay was performed to detect DNA strand breaks as described earlier [17]. Briefly, after 72 h of treatment with ASHD, (50 and 250 μM) cells were harvested, washed, mixed with low melting agarose (0.5%) and spread on agarose (1.5%) precoated slides. The cells were lysed, subjected to electrophoresis and stained with propidium iodide. The slides were observed and photographed under a fluorescence microscope (Olympus BX51 upright fluorescence Microscope, USA).

### Western blot analysis

To examine the expression levels of various proteins, Reh cells were harvested following treatment with ASHD (100 μM, 24, 48 and 72 h) and cell lysates were prepared as described earlier [17]. In the case of K562, cells were harvested after 48 h of ASHD treatment (30 μM) and used for cell lysate preparation. Equal amount of lysates (∼40 μg) were resolved on a SDS–polyacrylamide gel. The proteins were blotted onto a PVDF transfer membrane (Millipore, USA) and incubated sequentially with primary antibody and appropriate secondary antibody (Santa Cruz Biotechnology, USA). Primary antibodies against BAD, BCL2, BAX, procaspase 3, caspase 8, 9, cytochrome c, KU70, KU80, p-histone H3, PCNA, cyclin B1, CDK2, α-tubulin (Santa Cruz Biotechnology, USA), p53 and PARP (Calbiochem, USA) were used for current study. The protein expression was visualized by an enhanced chemiluminescence solution (Immobilon ^TM^ western, Millipore, India) and scanned by gel documentation system (LAS 3000, Fuji, Japan). Subsequently, blots were stripped and reprobed with anti-tubulin. All western blotting analyses were repeated a minimum of two times with good agreement.

### Effect of a pan-caspase inhibitor on ASHD treated Reh cells

The effect of a pan-caspase inhibitor, Z-VAD-FMK was studied [18] by incubating Reh cells with the inhibitor (50 μM) and ASHD (30 μM). Since ASHD and Z-VAD-FMK were dissolved in DMSO, cells with DMSO were used as control (equivalent to DMSO used in 30 μM concentration). Following treatment, the cells were stained with trypan blue and counted every 24 h. The viability of the cells was further verified using MTT assay following 24 and 48 h of treatment as described above.

### Statistical analysis

The results of cell viability studies were expressed as the mean ± standard deviation. The significance of differences between the experimental values was determined by GraphPad software using one-way ANOVA followed by Tukey-Kramer Multiple Comparison Test. A probability of p<0.001 and p<0.01 indicates statistically significant values.

## Results

### ASHD affects the viability of leukemic cells

As an initial screen for identifying the most potent derivative of azaspiro hydantoin molecules, various candidate molecules were tested to study their cytotoxicity in leukemic cells [13]. Based on this, ASHD (Propyl-2-(8-(3,4-difluorobenzyl)-2′,5′-dioxo-8-azaspiro[bicyclo[3.2.1] octane-3,4′-imidazolidine]-1′-yl)acetate) was identified as the most promising compound (Fig. 1A). Effect of ASHD on two leukemic cell lines, Reh (B-cell leukemia) and K562 (chronic myelogenous leukemia) were studied using MTT assay. ASHD was cytotoxic to both the cell lines used as shown by the reduced cell proliferation in MTT assay (Fig. 1B,C). The results showed that the IC_50_ value of ASHD was 28 µM, in case of both Reh and K562 cells, at 72 h of incubation (Table 1). In order to check the cytotoxicity of ASHD on normal cells, a human T-cell line, 8E5 was used. Results showed that the effect of ASHD on viability of 8E5 cells was significantly less as compared to Reh and K562 cells (compare, Fig. 1D with Fig. 1B,C) suggesting that ASHD is less toxic to normal cells as compared to cancer cells. IC_50_ value of ASHD on normal cells was estimated to be around 90 μM at 72 h (Table 1). Overall, the results showed that ASHD was a potent cytotoxic compound for leukemic cells.

**Table 1:** IC_50_ value of ASHD calculated at different time points.

Cell lines used	IC_50_ values ASHD (µM)	
	48 h	72 h
Reh	48	28
K562	38	28
8E5	76	87

### ASHD affects the proliferation of leukemic cells

**Figure 2:**
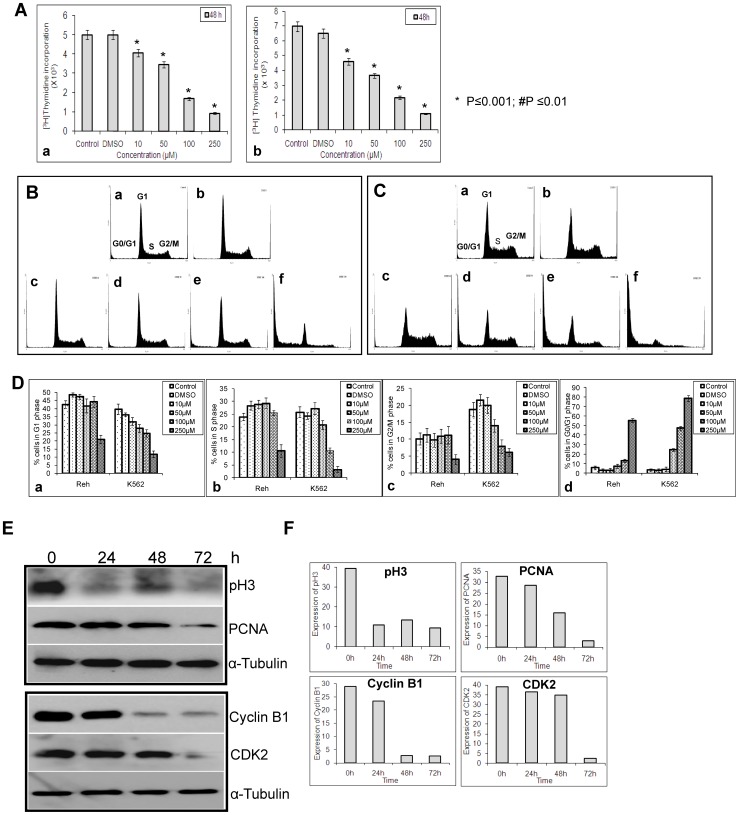
Effect of ASHD on cell cycle progression in leukemic cells. **A** Tritiated thymidine incorporation assay to determine the effect of ASHD on cell proliferation. The incorporation of tritiated thymidine was measured after 48 h of compound treatment. Incorporation of [^3^H] Thymidine after addition of ASHD to Reh (**a**) and K562 (**b**) cells following addition of ASHD. Error bars represented in each panel is based on three independent batches of experiments. **B–C**. Reh and K562 cells were harvested following incubation with ASHD (10, 50, 100 and 250 µM) for 72 h, stained with propidium iodide and sorted using FACS. Results are presented as histograms and are representative of two independent experiments with similar findings. Histogram resulting from the FACS analysis of Reh cells (**B**) and K562 cells (**C**). In each panel, **a** and **b** are histogram of control cells and cells treated with DMSO, respectively. **c**, **d**, **e** and **f** represent cells treated with ASHD (10, 50, 100 and 250 µM, respectively). **D**. Histograms showing the quantification of effect of ASHD treatment on specific cell cycle stages of Reh and K562. Comparison of G1 phase (**a**), S phase (**b**), G2/M phase (**c**) and G0/G1 phase (**d**) are shown. Error bars represented in each panel is based on two independent batches of experiments. **E**. Expression levels of cell cycle related proteins upon ASHD treatment. Western blotting studies showing expression profile of p-Histone 3, PCNA, Cyclin B1and CDK2 following treatment of Reh cells with ASHD (100 μM). **F**. The quantification of the western blots for each protein shown in panel E.

One of the common methods used for studying cell proliferation is based on the incorporation of tritiated thymidine into the DNA of dividing cells. Since ASHD affects the viability of the cells, we were interested in testing whether it affects cell cycle progression. Reh or K562 cells were cultured in the presence of [^3^H] thymidine following addition of ASHD. Results showed a reduction in the incorporation of [^3^H] thymidine, which was proportional to the concentration of the compound (10, 50, 100 and 250 μM) used (Fig. 2A,B). These results suggest that one of the mechanisms of induction of cytotoxicity by ASHD could be by interfering with DNA replication and thereby cell division.

### Effect of ASHD on cell cycle progression

In order to determine whether the growth inhibition by ASHD was due to a replication defect or apoptosis, FACS analysis was performed. For this, Reh and K562 cells were incubated with ASHD (10, 50, 100 and 250 μM, 72 h) and subjected to flow cytometry. The histogram of the control (Fig. 2B (a); C(a)) or DMSO (Fig. 2B (b); C(b)) treated cells showed a standard cell cycle pattern, which included G1 and G2/M peaks separated by S phase peak. Sub G1 peak (mostly dead cells) was absent. Upon addition of ASHD to Reh cells, a concentration dependent change was observed in the cell cycle pattern (Fig. 2B(c–f)). Comparable changes in the cell cycle pattern of ASHD treated cells were also noted in the case of K562 (Fig. 2C (c–f)). However, after ASHD treatment, the number of sub G1 (G0/G1) cells were more in K562 cells when compared to Reh cells (Fig. 2D (d)). Although we were not able to visualize a prominent arrest at any stage of the cell cycle, we noted that there is a transient block in S and G2/M phase up to 100 μM in the case of Reh treated cells (Fig. 2D (b & c)), which was not visible in the case of K562 cells (Fig. 2D). Quantitation of cell population in the sub-G1 phase indicated an increase in the number of dead cells in a dose-dependent manner (Fig. 2D (d)). Hence, these studies further suggest that growth inhibition could be mediated by a DNA replication defect followed by cell cycle arrest and apoptosis.

Since our data suggested that ASHD might interfere with DNA replication, we were interested in checking the status of proteins related to replication and G1/S transition. Cell lysates were prepared from Reh, after treating with ASHD (100 µM for 24, 48 and 72 h) and checked for the expression of PCNA and CDK2, the proteins required for replication and entry of cells to S phase, respectively. Results showed a reduction in the expression of PCNA following 48 h treatment with ASHD (Fig. 2E,F). A decline in expression of CDK2 after 72 h was also observed (Fig. 2E,F). In addition to this, ASHD treatment also led to a 10-fold decrease in the expression of cyclin B1 at 48 and 72 h, explaining the transient cell cycle arrest at G2/M phase (Fig. 2E,F). This is in correlation with the decline of cells at G1 phase at 72 h as noted in FACS analysis and cytotoxicity studies. Since p-histone H3 is a marker for cell division, its expression was also tested. The results showed that the levels of p-histone H3 reduced upon treatment with ASHD from 24 h onwards; however, expression was robust in the control (Fig. 2E,F). Further studies may reveal the exact mechanism of inhibition of DNA replication induced by ASHD.

### ASHD treatment leads to translocation of phosphatidyl serine to the outer membrane of the cells

**Figure 3:**
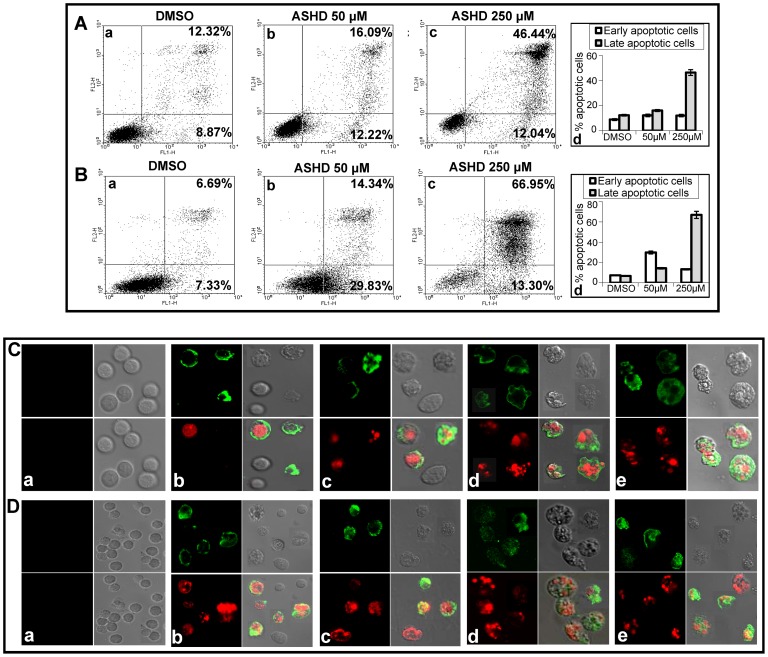
Detection of apoptosis induced by ASHD using flow cytometry and confocal microscopy. Reh (**A**) and K562 (**B**) cells were cultured with ASHD (50 and 250 μM) for 72 h and processed for Annexin V- FITC/PI double-staining. The cells were then quantitatively or qualitatively monitored. In panels **A** and **B**, lower left quadrant shows cells which are negative for both Annexin V-FITC and PI, lower right shows Annexin V positive cells which are in the early stage of apoptosis, upper left shows only PI positive cells which are dead, and upper right shows both Annexin V and PI positive, which are in the stage of late apoptosis or necrosis. The values mentioned in the quadrants show the percentage of cells positive for both the Annexin V and PI (Top) or Annexin V alone (Bottom). In both panels **A** and **B**, cells treated with DMSO (a), ASHD, 50 μM (b), and ASHD 250 μM (c) are shown. In both panels, bar diagram showing comparison of early and late apoptotic cells at different doses of ASHD treatment are presented (d). (**C**) and (**D**) shows confocal microscopy visualization of Reh or K562 cells, following treatment with ASHD. Cells incubated with DMSO alone (**a**), or ASHD 50 μM (**b, c**) and 250 μM (**d, e**) respectively are used for the study.

At lower doses of compound treatment, both cell cycle arrest and cell death were observed, while complete cell death was observed at higher doses. To test whether ASHD treatment led to cell death via apoptosis, Annexin V staining was carried out. Reh and K562 cells were harvested after 72 h of treatment with ASHD and used for Annexin V/PI double staining followed by FACS analysis. Dot plot results of ASHD treated Reh cells showed the presence of both early and late apoptotic cells (Fig. 3A). The effect was more pronounced when the experiment was performed on K562 cells (Fig. 3B). When DMSO treated cells were subjected to similar analysis (Fig. 3A, (a); 3B (a)), most of the control cells were negative for both Annexin V and PI, indicating that there was not much cell damage. Interestingly, we did not find cells stained with PI alone, indicating that ASHD treatment did not activate necrotic pathway. Thus, these results confirm the induction of apoptosis by ASHD at lower doses.

The Annexin V/PI double-stained cells indicate that such cells bear extensive cell membrane damage, which results in nuclear staining. This was further tested by confocal microscopy. The results showed that the control cells which were treated only with DMSO, did not show any staining with either Annexin V or PI, in the case of Reh or K562 (Fig. 3C(a) and D(a)). When cells were treated with 50 or 250 μM of ASHD, we could see cells stained by Annexin V-FITC (green colored rim) and intact nuclei, suggesting damage of cell membrane (Fig. 3C(b,c)) or stained by both Annexin V-FITC (green color) and PI (red color) suggesting complete damage of cell membrane (Fig. 3C, D, (b- e)). These results suggest a total disruption of cell membrane only when treatment was with higher dose.

### ASHD induces loss of mitochondrial membrane potential (Δψ_m_) in a dose- and time-dependent manner

**Figure 4:**
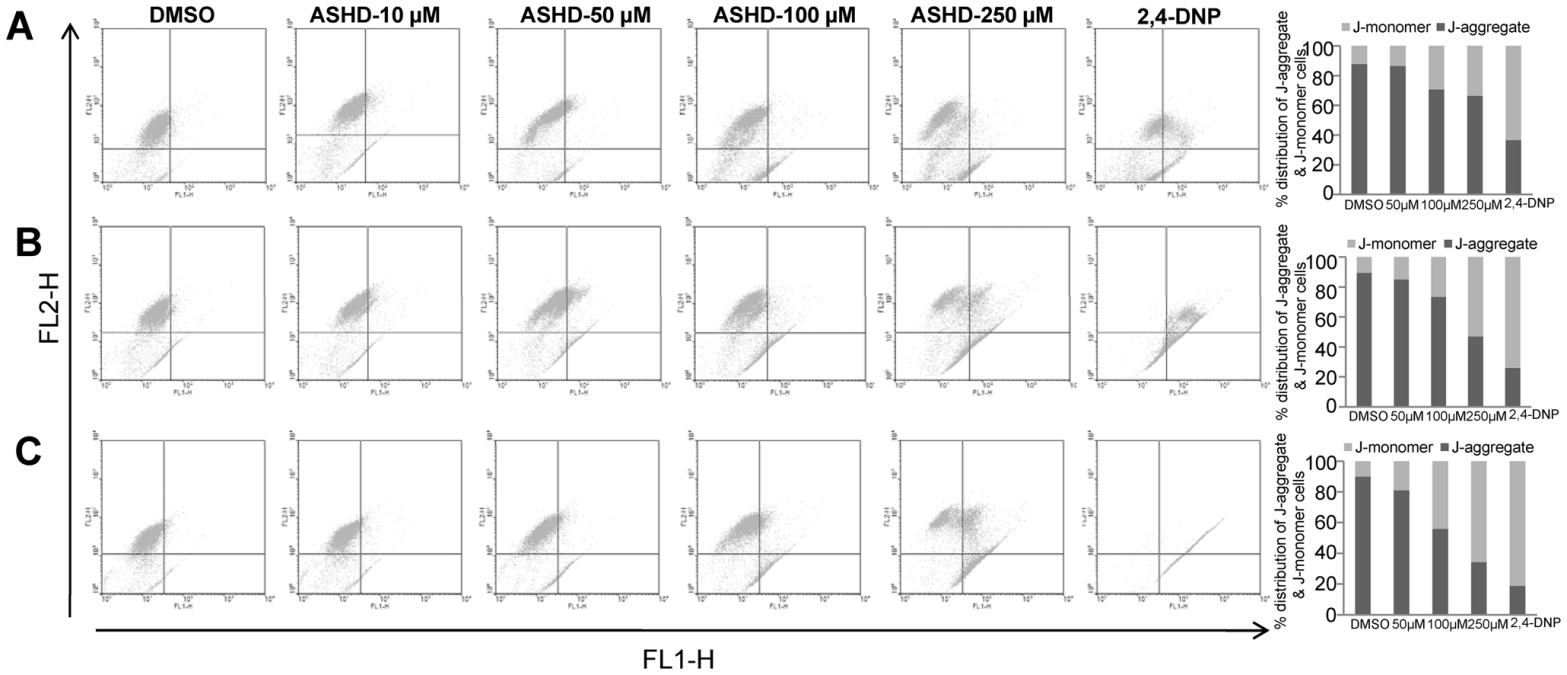
Effect of ASHD on mitochondrial transmembrane permeability (Δψ_m_). Reh cells incubated with ASHD at different concentrations for 24 (**A**), 48 (**B**) and 72 h (**C**) were stained with JC-1 dye and loss of mitochondrial membrane potential was assessed with the signal from monomeric and J-aggregate JC-1 fluorescence by flow cytometry. DMSO treated cells were taken as control. 2,4-DNP was used as a positive control. Dot plots show that Δψ_m_ increases in a dose- and time-dependent manner. Bar diagram showing percentage of apoptotic and nonapoptotic cells are shown on the right side of each panel.

Measurement of mitochondrial membrane potential is one of the methods used for studying the initiation of apoptosis. To explore the effect of ASHD on the mitochondrial membrane potential, Reh cells were cultured in presence of increasing concentrations of the compound (10, 50, 100 and 250 μM), harvested after 24, 48 and 72 h and used for flow cytometric analysis following staining with fluorescent probe JC-1. Representative scattergrams for red-FL2-H (indicator of an intact mitochondrial membrane potential) and green-FL1-H (indicator of loss of membrane potential) fluorescence are presented (Fig. 4A, B & C). Results showed that DMSO treated cells, mostly exhibited red fluorescence indicating an intact mitochondrial membrane potential (Fig. 4A, B & C, 1^st^ panel). Upon addition of ASHD, the number of cells showing green fluorescence increased with concentration and time. This suggests that ASHD disrupts the mitochondrial membrane potential in a time- and concentration-dependent manner (Fig. 4A, B & C), resulting in the cytosolic accumulation of monomeric JC-1, which is an indicator of apoptosis by activation of the intrinsic pathway.

### ASHD induces apoptosis through intrinsic mitochondrial pathway

**Figure 5:**
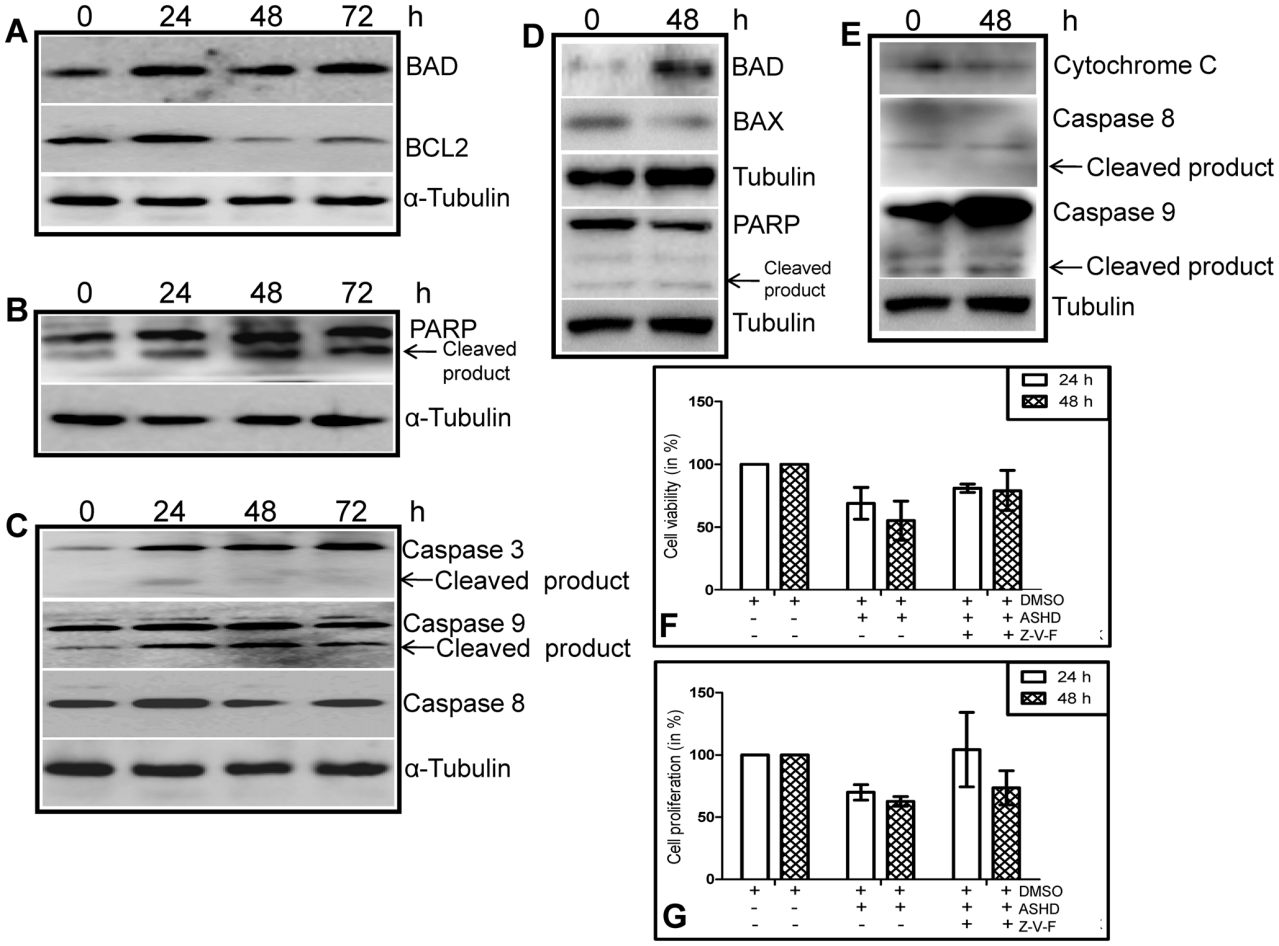
ASHD alters the expression of apoptotic proteins in Reh and K562 cells. **A–C**. Cell lysates were prepared from Reh cells after incubating with ASHD (100 µM) for 24, 48 and 72 h. DMSO treated cell lysate was used as control. Approximately, 40 µg of protein per sample was resolved on SDS-PAGE and transferred to a PVDF membrane. The membrane was probed for the expression of BAD, BCL2 (**A**), PARP (**B**) and caspase 3, caspase 9 and caspase 8 (**C**) with specific primary antibodies and appropriate secondary antibodies. **D–E**. Cell lysates were prepared from K562 cells after incubating with ASHD (30 µM) for 48 h and used for western blotting. The proteins studied are BAD, BAX, PARP (**D**), and caspase 8, caspase 9 and cytochrome C (**E**). The α-tubulin was used as an internal loading control in all the panels. **F–G**. Effect of pancaspase inhibitor (z-VAD-FMK) on Reh cells treated with ASHD. Approximately 0.75×10^5^ cells/ml were cultured and incubated with 30 µM ASHD, with or without 50 µM z-VAD-FMK. DMSO treated cells were used as vehicle control. **F**. MTT assay showing effect of ASHD on cell proliferation following treatment with z-VAD-FMK at 24 and 48 h. **G**. Trypan blue assay showing cell viability. For other details refer Fig. 1 legend. Error bars in panels, **F** and **G** are based on three independent batches of experiments.

To investigate the underlying mechanism by which ASHD induces apoptosis, we have studied the changes in the levels of proteins involved in apoptosis. Cell lysates were prepared from Reh cells treated with ASHD (100 μM) for 24, 48 and 72 h and used for western blot studies. Results showed that proapoptotic protein, BAD is upregulated immediately after 24 h of compound treatment (Fig. 5A). In contrast, the expression of the antiapoptotic protein, BCL2 was downregulated from 48 h onwards (Fig. 5A). However, it is interesting to point out that BCL2 expression was upregulated at 24 h of ASHD treatment. The results indicate that ASHD treatment disturbs the proapoptotic-antiapoptotic ratio within the cells, further confirming the activation of apoptosis.

Poly (ADP-ribosyl) polymerase (PARP) is a DNA repair enzyme that is downstream to caspase 3 in the apoptotic pathways. It is known that upon activation of the apoptotic pathway, caspase cleaves PARP into 85 and 27 kDa polypeptides. By immunoblot analysis using anti-PARP, we found that the addition of ASHD led to PARP cleavage in a time dependent manner, resulting in the accumulation of the 85 kDa product (Fig. 5B). Since PARP cleavage requires activation of caspases, we tested the level of active caspase 3 and caspase 9 in ASHD treated cells. Results showed that both caspase 3 and caspase 9 gets activated and cleaved upon addition of ASHD (Fig. 5C). Western blot analysis demonstrated that ASHD did not activate caspase 8 (Fig. 5C), which is required for the alternate death receptor-mediated apoptosis. Tubulin served as loading control in all the experiments (Fig. 5). Comparable results were obtained when BAD, PARP, caspase 8 and caspase 9 expression was studied in K562 cells following addition of ASHD (Fig. 5D–E). However, ASHD treatment in K562 cells led to the downregulation of BAX at 48 h (Fig. 5D). We could also observe a limited increase in cytochrome C release upon treatment with ASHD (Fig. 5E). Taken together, our results suggest that ASHD induces activation of proapoptotic proteins and downregulation of antiapoptotic proteins to induce the intrinsic pathway of apoptosis in leukemic cells.

### Pan-caspase inhibitor reverts ASHD mediated cell death

The effect of a pan-caspase inhibitor, Z-VAD-FMK on Reh cells treated with ASHD (30 μM of) was tested. Since ASHD and Z-VAD-FMK were dissolved in DMSO, cells treated with DMSO were used as control. The effect of ASHD on Z-VAD-FMK treated Reh cells was assessed using trypan blue and MTT assays. Cells were harvested after 24 and 48 h, stained with trypan blue and viable cells were counted. Results showed a partial protection on ASHD treatment upon treatment with pan-caspase inhibitor (Fig. 5F). The viability of the cells was further verified using MTT assay following 24 and 48 h of treatment. Cells treated with ASHD showed a decrease in viability to ∼60% when compared to the DMSO control; whereas cells treated with Z-VAD-FMK indicated a partial protection of cells against ASHD and showed an increase in cell viability almost equal to the DMSO control (Fig. 5G). Thus our results suggest that inhibition of caspases could revert the cytotoxicity induced by ASHD, further suggesting the activation of the intrinsic pathway of apoptosis upon treatment with ASHD.

### DNA strand breakage and altered expression of DNA repair proteins induced by ASHD

**Figure 6:**
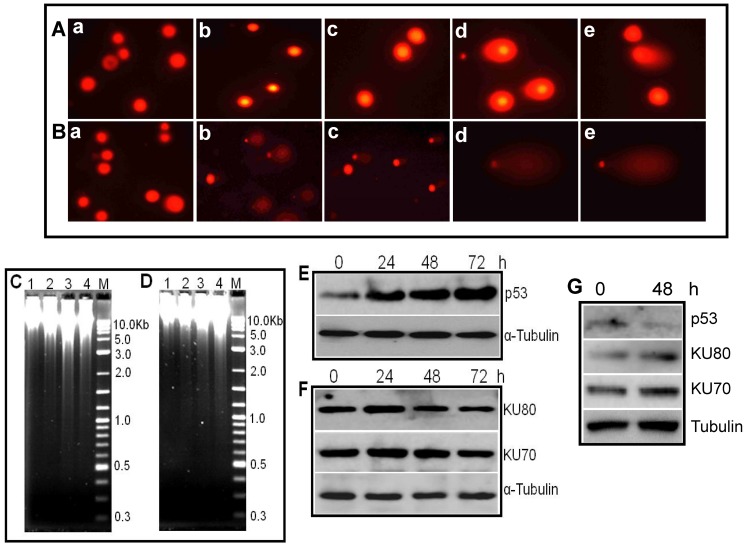
Detection of DNA strand breaks and repair proteins in Reh and K562 cells following treatment with ASHD. ASHD treated Reh (**A**) or K562 (**B**) cells (72 h) were subjected to DNA damage analysis by comet assay. In both panels, cells were treated with (a) vehicle control, (b, c) ASHD 50 μM and (d, e), ASHD 250 μM. (**C, D**). Agarose gel profiles showing DNA fragmentation. The chromosomal DNA was extracted from Reh (**C**) and K562 (**D**) cells, following treatment with different concentrations of ASHD. The purified DNA was then resolved on a 1% agarose gel. In both panels, Lane 1, DMSO, Lane 2–4, 50, 100 and 250 μM of ASHD, respectively. “M” is Marker. (**E–G**) Altered expression of p53 and KU70/80 following treatment with ASHD in Reh (**E,F**) and K562 (**G**) cells studied using immunoblotting. For other details refer Fig. 6 legend.

**Figure 7:**
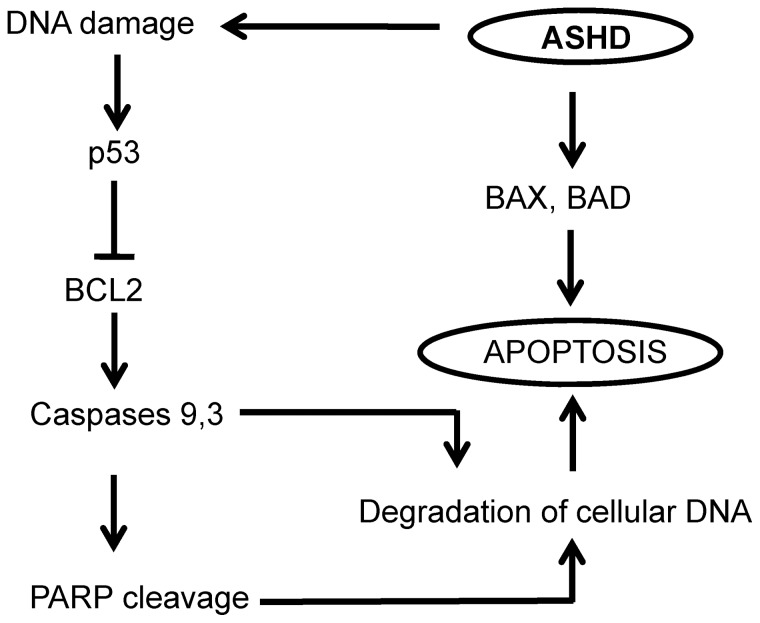
Proposed model for the mechanism of ASHD induced cytotoxicity through apoptosis. ASHD treatment can induce apoptosis through one of the following pathways. First, the DNA damages induced by ASHD could lead to the activation of p53, which can block the expression of BCL2. Downregulation of BCL2 probably leads to loss of mitochondrial membrane permeability and further to the activation of the initiator caspase 9. This results in the activation of the apoptotic protein cascade wherein caspase 3 gets cleaved and activated which can further either induce PARP cleavage or cleave the inhibitor of the caspase-activated DNase which finally results in fragmentation and degradation of the cellular DNA. In addition, ASHD activates p38 MAPK pathway, upregulation of proapoptotic proteins and BAD, which can directly result in apoptosis.

DNA fragmentation is a hallmark of apoptosis. The parameters assessed for DNA damage upon treatment with compounds were nuclear condensation and chromosomal DNA fragmentation. Reh and K562 cells were treated with 50 or 250 μM of ASHD for 72 h and used for single cell gel electrophoresis. Around 6–8 slides were examined from both vehicle control and treated samples. In case of Reh cells, we could mostly observe cells with nuclear condensation, upon treatment with ASHD (Fig. 6A). In contrast, when similar treatment was performed on K562 cells, we observed both condensation and formation of comets (Fig. 7B). Both nuclear condensation and comets were absent in Reh or K562 cells when treated with DMSO alone (Fig. 6A(a) and B(a)). These results indicate that ASHD treatment induced DNA damage in both leukemic cells, although the effect was more pronounced in the case of K562 cells. Further, DNA fragmentation followed by ASHD treatment was assessed by agarose gel electrophoresis of the genomic DNA from Reh and K562 cells (Fig. 6C,D). Consistent with the comet assay, the results showed increased DNA fragmentation upon treatment with increasing concentrations of ASHD. Thus, ASHD induce both fragmentation and condensation of chromosomal DNA, leading to apoptosis.

We have also tested the expression of master regulator p53, upon addition of ASHD. We found that ASHD induced activation of p53 in a time-dependent manner in Reh cells. We observed the induction as early as 24 h post-treatment of ASHD (Fig. 6E). KU70 and KU80 are DNA end binding proteins involved in nonhomologous end joining, a pathway involved in DNA double-strand break repair in mammalian cells. Hence, we were interested in testing the expression levels of both KU70 and KU80 upon treatment with ASHD. Following western blot analysis we found reduced expression of both the proteins (Fig. 6F). These results, further suggest that ASHD treatment leads to the downregulation of KU proteins and hence promotes apoptosis in Reh cells.

It is established that expression of p53 in K562 is very less [19]–[21]. Hence we were interested in testing its expression in K562 cells following treatment with ASHD (30 µM). Results showed that as reported earlier, the expression of p53 was very low, and ASHD treatment led to a minor difference in its expression (Fig. 6G). Interestingly we find that expression of both KU70 and KU80 was upregulated following treatment with ASHD in K562 cells (Fig. 6G), which is contradictory to that in Reh cells. The significance of this observation needs to be studied further.

## Discussion

In the present study, we observe that the spiro hydantoin derivative, ASHD, having an alkyl ester group has potent cytotoxicity against leukemic cells. Tritiated thymidine assay shows that ASHD is able to inhibit DNA replication leading to inhibition of cell growth. The concomitant decrease in the expression of CDK2 and PCNA was consistent with a block at S phase induced by ASHD. Since a transient arrest in S-G2/M was seen, one would expect an alteration in the level of expression of cyclin B1, a protein shown to have a role in cells entering into G2/M-phase. Western blot analysis showed that ASHD treatment led to the downregulation of cyclin B1. Hence, it appears that ASHD induces cell cycle arrest at least to a limited extent before triggering apoptosis.

Apoptosis is a fundamental process essential for both development and maintenance of tissue homeostasis [22]. One of the early events in apoptosis is translocation of phosphatidylserine (PS), which is normally confined to the cytoplasmic face of the plasma membrane, to the cell surface. The appearance of the sub-G1 population during cell cycle analysis confirmed the presence of apoptotic cells. Further, early and late apoptotic cells were quantitated by using Annexin V-FITC/PI double-staining. The morphological changes during apoptosis include membrane blebbing, cell shrinkage, chromatin condensation, DNA fragmentation and formation of apoptotic bodies. By performing confocal microscopy, comet assay and DNA fragmentation, we noted that ASHD treated cells showed all morphological changes characteristic of apoptosis.

In order to delineate the molecular details concerning cell death induced by ASHD, we investigated the possible molecular mechanisms involved [23]. Intrinsic pathway of apoptosis is one of the major pathways leading to cell death, which is regulated by BCL2 family of proteins. To test the role of these proteins in ASHD induced cell death, we measured their expression levels. It was noted that the levels of proapoptotic protein, BAD were upregulated, while those of antiapoptotic protein, BCL2 were downregulated leading to an increase in the ratio of proapoptotic to antiapoptotic proteins which has also been shown by others. Activation of caspases is the hallmark of apoptosis [24]. We have noted that both caspase 3 and caspase 9 are activated and cleaved. PARP cleavage is shown to have a role in DNA damage induced apoptosis and is the major target of caspase 3 [25]. We found that following ASHD treatment, caspase 3 activation leads to PARP cleavage. Further, we find that treatment of a pancaspase inhibitor on cells pretreated with ASHD resulted in a partial block of ASHD mediated cell death. These observations indeed suggest that ASHD triggers the activation of intrinsic pathway of apoptosis.

If ASHD mediated cell death occurs through the intrinsic pathway of apoptosis, one would anticipate a change in the mitochondrial membrane potential leading to the release of cytochrome C. Our studies using JC-1 staining conjunction with the western blotting indicated a loss of mitochondrial membrane potential, further supporting the activation of the intrinsic pathway. Thus, our results are in accordance with the earlier observations that the high ratio of BAD to BCL2 could lead to loss of mitochondrial membrane potential and apoptosis.

We have noted that the status of p53 is not same in the two cell lines, Reh and K562 studied, although expression of many apoptotic markers was comparable. Reh cell line express p53, whereas, K562 being a p53 deficient cell line express p53 only to a very low level [19]–[21] and hence may be more vulnerable to ASHD. Quantitative increase in p53 with the time of drug treatment indicates that the apoptotic pathway maybe controlled by p53 in Reh. But in the case of K562 which is a p53 null cell line, the drug may cause its effect through other alternate pathways, which need to be investigated.

In summary, ASHD induces DNA damage and downregulates BCL2, leading to the loss of mitochondrial membrane potential (Fig. 7). ASHD activates caspase 9, which in turn results in the activation of caspase 3. This leads to the cleavage of PARP, which hampers its ability to carry out poly-ADP-ribosylation of various proteins involved in DNA repair such as KU70 and KU80, causing the failure of repair of damaged DNA, which in turn leads to degradation of cellular DNA. Simultaneously, caspase 3 could activate nucleases, which will also cause degradation of the nuclear DNA leading to apoptosis (Fig. 7). However, more studies are required to unravel the signaling mechanism induced by ASHD. Hence, the present study provides a new insight on spiro hydantoin derivatives serving as potential therapeutic agents for leukemia.
